# Skin Matters: Identifying Pain Mechanisms and Predicting Treatment Outcomes

**DOI:** 10.1155/2013/329364

**Published:** 2013-05-21

**Authors:** Edward A. Shipton

**Affiliations:** Department of Anesthesia, University of Otago, P.O. Box 4345, Christchurch 8041, New Zealand

## Abstract

The skin acts as a complex sensory organ. The emerging new data on peripheral pain mechanisms from within the skin is presented. This data has led to new insights into the potential pain mechanisms for various pain conditions including neuropathic pain (from small fiber neuropathies) and Complex Regional Pain Syndrome. The somatosensory neurons that innervate our skin constantly update our brains on the objects and environmental factors that surround us. Cutaneous sensory neurons expressing nociceptive receptors such as transient receptor potential vanilloid 1 channels and voltage-gated sodium channels are critical for pain transmission. Epidermal cells (such as keratinocytes, Langerhans cells, and Merkel cells) express sensor proteins and neuropeptides; these regulate the neuroimmunocutaneous system and participate in nociception and neurogenic inflammation. In the past two decades, there has been widespread use of modalities such as punch skin biopsies, quantitative sensory testing, and laser-evoked potentials to evaluate small caliber nerve fibers. This paper explores these laboratory techniques as well as the phenomenon of small fiber neuropathy. Treatment using transdermal drug delivery is discussed. There is potential for these findings to predict treatment outcomes in clinical practice and to develop new therapies for different pain conditions. These findings should enhance the physician's ability to evaluate and treat diverse types of pain.

## 1. Introduction

The skin has homeostatic and immunologic barrier functions, but acts as a complex sensory organ as well [1]. The somatosensory neurons that innervate our skin constantly update our brains on the objects and environmental factors that surround us [2]. The neuroimmunocutaneous system (NICS) is responsible for the cutaneous sensations of touch, pressure, temperature, and pain. This sensory transduction occurs via primary afferent nerves following reciprocated signals between neuronal and nonneuronal skin cells of the NICS [1]. New data concerning peripheral pain mechanisms from within the skin have led to new insight into the potential pain mechanisms for various pain conditions including neuropathic pain syndromes such as diabetic neuropathy and Complex Regional Pain Syndrome.

### 1.1. Cells and Channels

**Figure 1:**
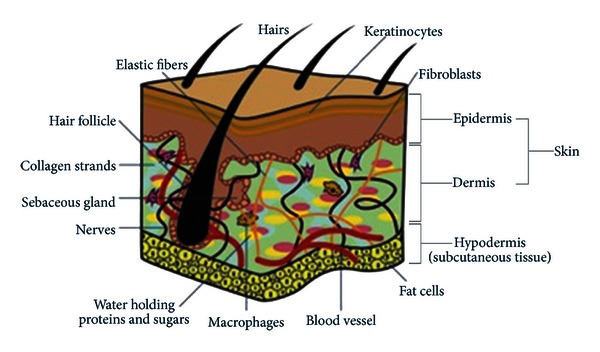
The skin layers.

The epidermis is largely composed of multiple layers of keratinocytes along with melanocytes, Langerhans cells, and Merkel cells (Figure 1) [3]. All these epidermal cells express sensor proteins and neuropeptides that regulate the NICS and participate in nociception and neurogenic inflammation. Keratinocytes form tight junctions with sensory afferent nerves [1]. Keratinocytes (that account for 85% of the cells of the dermis) have a close anatomic relationship with peripheral nerves; they have transient receptor potential vanilloid 1 (TRPV1) channels and transient receptor potential ankyrin 1 (TRPA1) channels on their surface [1, 4]. Keratinocytes may also contribute to human pain states via elevated Na^+^ (V) channel expression in Complex Regional Pain Syndrome (CRPS) and in postherpetic neuralgia (PHN) [5]. 

Cutaneous sensory neurons expressing nociceptive receptors such as transient receptor potential vanilloid 1 [TRPV1] channels and voltage-gated sodium [Na^+^ (V)] channels are critical for pain transmission [1]. The TRPV1 and TRPA1 channels are members of the transient receptor potential (TRP) superfamily of structurally related, nonselective cation ion channels [4]. TRPV1 and TRPA1 channels interlink with each other [4]. The transient receptor potential vanilloid type 1 (TRPV1) is a noxious heat-gated cation ion channel activated by exogenous irritants, including protons and several endogenous lipid ligands; these include endocannabinoids, lipoxygenase metabolites, lysophosphatidic acid and oxidized linoleic acid metabolites released either in response to noxious heating or under pathological pain conditions [6]. 

Stimulation of the TRPV1 channels allows the influx of calcium and sodium ions resulting in depolarisation and the possible generation of an action potential transmitted as excitatory signals to the central nervous system [7]. Thus the TRPV1 channel serves as a sensory transducer ion channel in nociceptive nerve endings for signalling various painful stimuli [6]. It is highly expressed on nociceptive sensory nerves, as well as on nonneuronal skin cells, especially keratinocytes [1]. Other non-neuronal cells on which TRPV1 and TRPA1 channels are expressed are vascular smooth muscle and endothelium [4]. 

In pain and neurogenic inflammation, TRPV1 is coexpressed on TRPA1-expressing sensory nerves; both integrate a variety of noxious stimuli [4]. Complex signaling pathways between cells of the NICS, such as keratinocytes, TRPV1-expressing nociceptors, and macrophages, lead to the release of neural growth factor (NGF), prostaglandins, opioids, proinflammatory cytokines, and chemokines [1]. These lead to sensitisation of the peripheral nerves by upregulating ionic channels and by inducing further spinal cord cytokine release [8]. 

Sodium channel isoforms preferentially expressed in the peripheral nervous system, including Na^+^ (V) 1.3, 1.9, and especially 1.7 and 1.8, play specific roles in the neurobiology of pain [9]. 

## 2. Small Fiber Neuropathy (SFN)

Neuropathic pain arises as a direct consequence of a lesion or disease of the somatosensory system; it affects about 7% of the general population [10, 11]. Small nerve fibers have been traditionally considered invisible, as they could not be detected by routine nerve conduction study. Small fiber neuropathy is a neuropathy of the small nonmyelinated fibers and myelinated A delta fibers. Neuropathic pain occurs from small fiber neuropathy; small fiber neuropathy is caused by a wide variety of acquired and genetic disorders [12], many of which are treatable [13]. Small fiber neuropathy has been considered the prototype of painful neuropathy. However, there are no studies providing conclusive data on the prevalence and incidence of SFN [12]. 

### 2.1. Aetiology

Diabetes mellitus is the most frequent underlying cause of SFN [14]. Other causes include toxic (e.g., alcohol), metabolic, immune-mediated, infectious, and hereditary causes. Erythromelalgia is a clinical syndrome distinguished by the occurrence of red, hot extremities. There is increasing recognition that most adult patients have electrophysiologic evidence of an associated small-fiber neuropathy [15]. Studies have shown that mutations in *SCN9A*, which encodes the sodium channel protein Na^+^ (V) 1.7 subunit, are present in some patients with inherited erythromelalgia, but are rare in noninherited cases of erythromelalgia [16]. 

The relationship between loss of intraepidermal nerve fibers (IENF) degeneration and neuropathic pain led to the discovery that SFN can be caused by mutations in sodium channels [17]. In 29% of a group of patients with idiopathic small fiber neuropathy, a SCN9A gene mutation encoding for Na^+^ (V) 1.7 sodium channels was recently identified [14]; this mutation leads to hyperexcitability of the dorsal root ganglion neurons [14]. In humans, gain-of-function mutations in *SCN9A*, which encodes Na^+^ (V) 1.7, lead to severe neuropathic pain, whereas loss-of-function mutations in this gene lead to indifference to pain [18]. 

### 2.2. Symptoms and Signs

About 60% of patients describe the painful sensation as spontaneous (burning, sunburn-like, paroxysmal, pruritic, and deep), with worsening at rest or during the night [12]; the sensation can be associated with thermal evoked pain (cold or warm) with or without allodynia, a painful response to a normally innocuous stimulus, and hyperalgesia, an increased response to a painful stimulus [12]. In addition there are negative sensory symptoms (thermal and pinprick hypoesthesia) that reflect peripheral deafferentation [19]. Sensation of cold feet is reported, though warm to touch. Thermal hypoesthesia with or without pinprick hypoesthesia has been detected in 40% of patients [20]; hyperalgesia and aftersensation have been detected in 10–20% of patients [12, 20]. 

Additional autonomic dysfunction (mediated by skin cholinergic and vasomotor fibers) leads to frequent vascular deregulation in lower limbs [12, 20]. Other autonomic dysfunctions include sweating abnormalities [20–22]. Most patients with SFN have abnormal pinprick and light touch sensations, although more than a third have a normal sensory examination [20, 21]. 

### 2.3. Complex Regional Pain Syndrome (CRPS)

CRPS is a syndrome characterized by a continuing (spontaneous and/or evoked) regional pain, that is, seemingly disproportionate in time or degree to the usual course of any known trauma or other lesion [23]. The pain is regional (not in a specific nerve territory or dermatome); it usually has a distal predominance of abnormal sensory, motor, sudomotor, vasomotor, and/or trophic findings. It has signs of central sensitisation such as allodynia and hyperalgesia. The syndrome shows variable progression over time [23].

Elevated proinflammatory cytokines (systemically and locally), increased neurogenic inflammation, and autoantibodies all play a part in the pathophysiological development of CRPS [24]. Accumulating experimental and clinical evidence supports the hypothesis that Complex Regional Pain Syndrome type I (CRPS-I) might indeed be a small fiber neuropathy [25]. Most post-traumatic inflammatory changes observed in CRPS are mediated by two peptides, CGRP and substance P [26]. The activation of cutaneous nociceptors can induce retrograde depolarisation of small-diameter primary afferents, causing release of neuropeptides such as substance P and CGRP from sensory terminals in the skin. These neuropeptides evoke vasodilation and protein extravasation in the tissue [26]. The resulting signs (reddening, warming, and oedema) are labeled neurogenic inflammation [26]. Tumour necrosis factor-alpha (TNF-alpha) has been found to be high in skin samples from CRPS patients compared to samples from patients with fractures without CRPS [27]. 

Tactile afferents terminate in morphologically specialized end organs that govern their mechanosensory responses and allow them to extract distinct features of a complex tactile stimulus [2]. Disturbances in body perception (such as tactile acuity) are increasingly acknowledged as a feature of CRPS. Understanding the relationship between body perception disturbance, pain, and tactile acuity might provide insight into alternative avenues for treatment [28]. 

## 3. Skin Laboratory Tools

Standard neurophysiological responses to electrical stimuli (nerve conduction studies, and somatosensory-evoked potentials) do not assess the function of nociceptive pathways [29, 30]. There are other laboratory tools for assessing neuropathic pain using the skin in human patients.

A specific diagnostic test for small fiber neuropathy is a skin biopsy; this includes a count of the intraepidermal small nerve fibers (IENF) that cross the basal membrane. The loss of IENF can be reliably measured and is currently used to diagnose small fiber neuropathy (SFN) [17]. Another biopsy is blister biopsy used mainly in CRPS; it is based on the suction of the epidermis alone [31]. It does not cause bleeding, and there is no need for local anesthesia [12]. Other tests include quantitative sensory and autonomic testing [14]. 

### 3.1. Skin Biopsy

**Figure 2:**
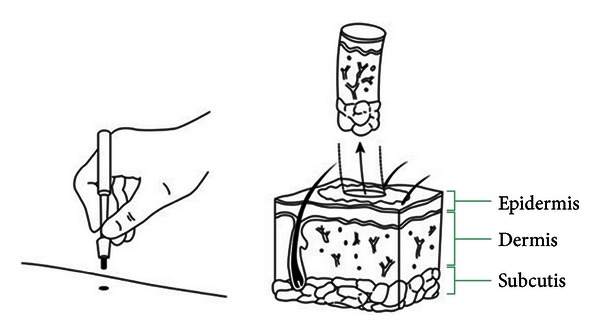
Punch skin biopsy.

In the past two decades, there has been widespread use of punch skin biopsies and quantitative sensory testing to evaluate small caliber nerve fibers [13]. Skin biopsy has emerged as a novel tool that readily permits morphometric and qualitative evaluation of somatic and autonomic small nerve fibers (Figure 2) [19].

Antibodies against protein gene product (PGP) 9.5, a form of ubiquitin carboxyl-terminal hydrolase, are commonly used to mark nerve fibers [29]. Bright-field immunohistochemistry and indirect immunofluorescence with or without confocal microscopy form the two immunostaining methods most commonly used [29]. Immuno-staining labels the different structures, such as nerve fibers, sweat glands, blood vessels, and resident or infiltrating cells. The availability of commercial tests for SFN has enabled clinicians to easily measure IENF density.

IENF density has proved to be the most reliable technique to diagnose SFN [12, 32]. There are recently available age-adjusted and sex-adjusted normative values for intraepidermal nerve fiber density [12, 33]. A task force of the European Federation of Neurological Societies and the Peripheral Nerve Society has stated that a reduced IENF density is associated with the risk of developing neuropathic pain [34]. The density has correlated with the loss of pinprick sensation in idiopathic SFN [12, 35]. In HIV neuropathy, the density has predicted the risk of developing neuropathy symptoms over a 2.9-year period [12, 36]. 

Skin biopsy is much less invasive and more practical than peripheral nerve biopsy. It is a safe and reliable tool for investigating nociceptive fibers in human epidermis and dermis [29]. It can be performed at any site of the body, with a disposable punch, using a sterile technique, and under local anesthesia (Figure 2) [29]. Obtaining biopsies from both distal (e.g., 3 mm at 10 cm above the lateral malleolus within the territory of the sural nerve) and proximal sites in an affected limb can indicate whether an SFN is length dependent or not [21]. Patients with SFN, that is, non-length-dependent are more likely to suffer from diabetes, impaired glucose tolerance, or an immune-mediated process as the cause for their neuropathy [21, 37–39]. 

Skin biopsies have been used to investigate epidermal nerve fibers in diabetic neuropathy [40] and in infectious and inflammatory neuropathies [41]. In all studies, epidermal nerve fiber density was significantly lower in patients with neuropathy than in controls [29]. Although examination of a skin biopsy invariably discloses reduced epidermal nerve fiber density in patients with painful neuropathy, it can very occasionally do so in patients with nonpainful neuropathy as well [29]. 

A recent study assessed the usefulness of skin biopsy in the assessment of 145 patients with suspected SFN [21]. In 59% of patients skin biopsy was abnormal in at least one site [21]. Patients with confirmed SFN were significantly more likely to have pain; they were more than twice as likely to respond to standard neuropathic pain medications [21]. A positive response to neuropathic pain medications was seen in 84% of patients with an abnormal skin biopsy compared to only 42% of those with a normal biopsy [21]. Skin biopsy has a relatively high yield in patients with sensory symptoms with no findings of mixed fiber neuropathy on clinical examination or on nerve conduction studies [21]. 

In CRPS patients skin biopsies show that the levels of TNF alpha are greater in the affected than the unaffected limb [27]. This difference resolves only gradually over approximately six years after injury [42]. 

### 3.2. Blister Biopsy

CRPS is associated with the presence of a proinflammatory state in blister fluid [43]. In chronic CRPS, significant increases were found in interleukin-1 receptor antagonist (IL-1Ra), monocyte chemoattractant protein-1 (MCP-1), macrophage inflammatory protein-1 beta (MIP-1*β*), and interleukin-6 (IL-6) in the blister fluid obtained [43]. In the CRPS-affected limb, the pooled estimates demonstrated significantly increased concentrations with large effect sizes for IL-1Ra and MCP-1, a moderate effect size for MIP-1[beta], and a small effect size for IL-6 [43]. 

In an observational study in patients with cold CRPS in one extremity, it was found that the fluid of artificially created skin blisters showed an imbalance between the vasodilator substance nitric oxide and the vasoconstrictive substance endothelin-1 [44]. This imbalance can be an outcome of endothelial dysfunction that can result from inflammatory disease. Besides the autonomic disturbances, this imbalance might provide another explanation for the diminished blood flow in cold CRPS [44]. 

### 3.3. Quantitative Sensory Testing (QST)

**Figure 3:**
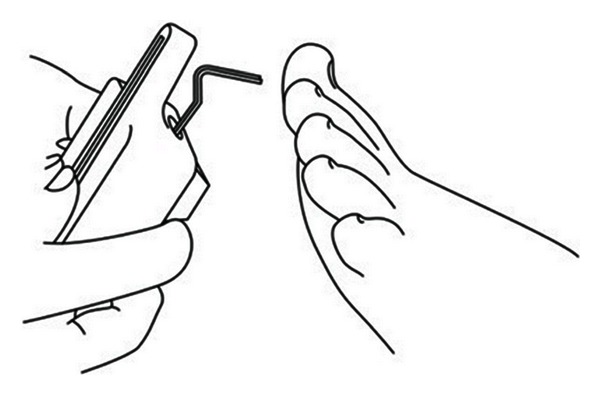
Quantitative sensory testing with electronic vibrameter.

Quantitative sensory testing is a physiological measure of perception in response to mechanical, thermal, and painful stimuli of controlled intensity [29, 45, 46]. Mechanical sensitivity for tactile stimuli is measured with plastic filaments that produce graded pressures (von Frey hairs), pinprick sensation (weighted needles), and vibration sensitivity (electronic vibrameter) (Figure 3). Thermal perception and thermal pain are measured using a thermode, or using other devices that operate on the thermoelectric effect [29, 45, 46]. 

QST is useful in the early diagnosis of diabetic neuropathy [45, 46], and for quantifying mechanical and thermal allodynia and hyperalgesia in painful neuropathic syndromes [29]. It is not specific for neuropathic pain, as QST changes can occur in nonneuropathic pain states such as rheumatoid arthritis or inflammatory arthromyalgia as well [29]. 

### 3.4. Laser-Evoked Potentials (LEPs)

LEPs are an easy and reliable technique for assessing nociceptive pathway function. Laser-generated radiant heat pulses selectively provide a rapid, synchronous, and selective activation of A-delta and C-thermosensitive nociceptors in the skin, without any concomitant activation of mechanoreceptors and A-beta fibers [47]. They accurately and objectively reflect the somatosensory transmission within the spinothalamic pain and temperature pathways [47]. 

LEPs are used to assess damage to the peripheral and central nociceptive system in peripheral neuropathies, syringomyelia, Wallerian degeneration, and multiple sclerosis [29]. LEPs are abnormal in conditions that engender structural damage, such as herpes zoster, tumour compression, and multiple sclerosis [29]. 

## 4. Transdermal Treatment of Peripheral Neuropathic Pain

Transdermal drug delivery is one of the most patient compliant routes of drug administration. Direct targeting of the skin with topical therapies should therefore be considered as a relevant treatment option for patients with peripheral neuropathic pain.

However, the stratum corneum, the outermost layer of the skin, resists the penetration of drugs across the skin. Hydrophilic, ionized, and macromolecular substances are poorly permeable across the skin [48, 49]. To enhance drug permeation in a passive manner, transdermal drugs should be lipophilic and should ideally have a molecular weight less than 500 Daltons [48, 50]. Alternatively energy dependent active measures can be used to enhance drug delivery across the skin. These include physical permeabilisation of skin or driving the drug molecule across the skin. In addition to the use of an eutectic mixture of local anesthetics and the use of controlled heat, other methods such as iontophoresis, electroporation, sonophoresis, and magnetophoresis can be used [48, 49]. 

### 4.1. Transdermal Local Anesthetics

Local anesthetics block axonal conduction by entering the nerve, where they bind to the sodium channel on the cytoplasmic side [51]. The addition of capsaicin allows opening of the pore size of the TRP channel large enough to allow entry of local anesthetics [52]. Topical delivery systems for local anesthetics are characteristically composed by a diversity of formulations (viscosity inducing agents, preservatives, permeation enhancers, and emollients) and presentations such as semisolid (gel, creams, and ointments), liquid (emulsions and dispersions), and solid (patches) pharmaceutical forms [51, 53]. The proposed formulations aim to reduce the local anesthetic concentration used, increase its permeability and absorption, keep the local anesthetic at the target site for longer and decrease the clearance, and limit local and systemic toxicity [51, 53]. 

Transdermal cream containing ketamine and lignocaine has proved effective in 73% of patients with acute neuropathic pain [54]. A topical lignocaine 5% w/w patch has demonstrated efficacy in postherpetic neuralgia [1, 55, 56]. Topical lignocaine is the most commonly prescribed treatment for erythromelalgia [15]. Only class III evidence supports its use for diabetic neuropathic pain [57]. 

### 4.2. Transdermal Capsaicin

The new dermal application system containing high concentration (8% capsaicin w/w) causes nociceptor desensitisation by continuous activation of primary sensory TRPV1-expressing neurons by the high doses of capsaicin over 30–60 minutes [58]. Reversible axonal degeneration with topical capsaicin occurs in healthy volunteers [59]. A recent systematic review found that high-concentration topical capsaicin used to treat post herpetic neuralgia and HIV neuropathy gave higher levels of pain relief than the control treatment using much lower concentrations of capsaicin [60]. 

### 4.3. Transdermal Dimethyl Sulphoxide

Transdermal treatment using the free radical scavenger dimethyl sulphoxide (DMSO) remains unsubstantiated in the treatment of CRPS [61]. Overall effects, however, appear positive [61]. 

### 4.4. Transdermal Nonsteroidal Anti-Inflammatory Drugs (NSAIDs)

Modern discoveries of peripheral mechanisms involved in the pathophysiology of neuropathic pain have justified the usage of NSAIDs in patients in this condition [62]. Nerve injury stimulates the release of phospholipids, which in turn activate phospholipase A2 and generate prostaglandin E2 [62]. This product binds to primary nociceptive fibers that phosphorylate sodium channels, transmitting the pain signal to the central nervous system. This peripheral sensitisation mediated by prostaglandins in primary afferent peripheral nociceptors could be blocked by NSAID topical agents [62]. Topical medications such as indomethacin, aspirin, and diclofenac have been used for neuropathic pain, despite inconsistent results [63].

### 4.5. Transdermal Clonidine

In painful diabetic neuropathy, pain may arise from sensitized-hyperactive cutaneous nociceptors. This abnormal signaling may be reduced by topical administration of the alpha-(2)-adrenergic agonist, clonidine, to the painful area. A recent randomized controlled study showed topical clonidine gel to significantly reduce the level of foot pain in painful diabetic neuropathy subjects with functional (and possibly sensitised) nociceptors in the affected skin (as revealed by testing with topical capsaicin) [64]. 

### 4.6. Tactile Discrimination in CRPS

In CRPS aberrant central processing has been suggested as the neural correlate of body perception disturbance and tactile impairment [28]. In patients with CRPS, sensory discrimination training increases tactile acuity, normalizes cortical reorganization, and decreases pain [65, 66]. 

## 5. Conclusion

Along with neuronal and immunological systems, the skin plays a critical role in sensory transduction [1]. Further direct targeting of the skin with topical agents should be considered. The interaction of TRPV1 and TRPA1 channels in the skin in painful conditions needs further exploration. Second generation TRPV1 antagonists (without on-target side effects of hyperthermia and burn risk) are under development [6]. 

Laboratory tools are used to help diagnose neuropathic pain and to quantify damage to the nociceptive pathways. New diagnostic criteria and normative values for skin biopsy have made the diagnostic approach more reliable in clinical practice; this will assist the quality of data obtained in future clinical trials [12]. Mutations in SCN9A encoding for Na^+^ (V) sodium channel have explained otherwise idiopathic SFN; this should hopefully prompt studies with selective sodium channel blockers in the therapy of neuropathic pain [9, 12]. Enhancing drug delivery across the skin for the treatment of peripheral neuropathic pain should be further investigated.

The use of skin biopsies to understand the sequence of events occurring after nerve degeneration in chronic neuropathies will promote better understanding of the ability of skin nerves to regenerate and the reasons for their failure [17]. There is potential for these findings to help predict treatment outcomes in clinical practice and, as a result, develop new therapies for different clinical pain conditions. This should enhance the physician's ability to evaluate and treat diverse types of pain. In Pain Medicine, the skin does indeed matter!
